# Evaluating the utilization of technological factors to promote e-commerce adoption in small and medium enterprises

**DOI:** 10.1007/s10660-023-09692-7

**Published:** 2023-03-28

**Authors:** Md Billal Hossain, Nargis Dewan, Aslan Amat Senin, Csaba Balint Illes

**Affiliations:** 1 Doctoral School of Economic and Regional Sciences, Hungarian University of Agriculture and Life Sciences, Godollo, 2100 Hungary; 2 Doctoral School of Business Administration, Kumoh National Institute of Technology, Gumi, 39177 South Korea; 3 Azman Hashim International Business School, University of Technology Malaysia, 81310 Skudai, Malaysia; 4 Hungarian National Bank—Research Center, John Von Neumann University, Kecskemet, 6000 Hungary

**Keywords:** SMEs, e-Commerce adoption, Technological factors, Bangladesh

## Abstract

This study examined the technological factor's utilization to promote the e-commerce in small and medium sized enterprises in Bangladesh. The technological factors are, Information and communication adoption, Internet connectivity, and Business data management. The e-commerce adoption factors involve the strategic innovation, research and development activity, and Productivity growth. The electronic commerce is being widely adopted in Bangladesh in the time of covid-19 pandemic. In the adoption of e-commerce, prominent factors are the internet connectivity, which plays a major role and Business data management which has equal importance for SMEs. SMEs have limited access to data as compared to multinational industries. However, the data has equal importance for multinational and SMEs to improve the e-commerce business. The research collects data from 115 SMEs from Bangladesh relating to different fields. The random selection method has been advised to collect data from SMEs aged not less than five years. The data collected for this research is through the interested parties of small and medium sized enterprises in Bangladesh. The data of the research is interpreted and analyzed through statistical software SPSS.

## Introduction

Technological factors are important to any organization, and their role is indispensable in business. Technological factors are essential to compete with the modern trends of modern society. In this research, the technological factors include ICT adoption, Internet connectivity, and business data management. There are ample theories about the technological factors and their utilization processes for small and medium-sized enterprises. This study is based on the technology acceptance model (TAM) [34]. Davis [35] proposed the technology acceptance model, and its second model, TAM2, is based on the theory of reasoned action and innovation diffusion theory [54]. TAM works as a general theory, which has many bases according on the industry's needs [34]. Other theories are,the theory of planned behavior (TPB [11], Unified Theory of Acceptance and Use of Technology (UTAUT), Diffusion of Innovation (DOI) [44], and the Technology-Organization-Environment (TOE) framework.

The role of information and communication technology adoption (ICT) is important in the use of businesses. It can be used for finding, developing, and analyzing present and persistent information, problems, and solutions to business-oriented issues. ICT adoption provided a rapid mechanism to access experiences and ideas from the global community and learn and experience people from different cultures and communities [17]. Information and communication technology adoption has become a powerful source of inspiration in society. A country's economic growth depends on small and medium-sized enterprises (SMEs). Small and medium-sized enterprises work in two ways, producers and users of information and communication technology, in the form of rapid productivity and easy and rapid communication to reach new clients.

Technological factors are the most powerful communication tools for competitive purposes inside organizations [3]. With time, organizations have developed a speedy information gathering, communication, and problem-solving process. As a result, ample web applications and software are available on the market to facilitate organizations. These web applications work on hardware such as computers, laptops, tablets, and other gadgets [19]. These technological advancements have made the decision-making process much easier for SMEs, and their routine tasks have become a click away.

With technological advancement, the model-making and concept-designing processes have become easier to find faults in computer programs. That has saved labor, time, and money for SMEs. Each small and medium-sized enterprise uses different ICT adoption tools differently. The internet connectivity in SMEs is based on 3G, and 4G broadband, which further connects with virtual private networks in the organization [1]. However, in Bangladesh, not all SMEs use high-speed internet connectivity. In several areas, the internet connectivity is limited to 3G only. However, the fastest internet connectivity available in the country is 5G [1].

Technological factors are important in adopting e-commerce in small and medium-sized firms. The role of internet connectivity is obvious, and no firm can adopt e-commerce without internet connectivity [21]. The non-availability of the internet leads a firm not to adopt e-commerce. In some cases, it has been observed that SMEs struggle to manage their secure internet availability. Still, the lack of infrastructure, poor circumstances, and environmental issues are hurdles to e-commerce adoption. Research and development are the keys to launching an e-commerce business. SMEs have acquired R&D activity through proper learning, but lack of implementation is the barrier to e-commerce adoption for SMEs in Bangladesh [2]. The R&D is fixed to focus on the local level, ignoring the national and international competitors. The bigger the data, the fewer issues SMEs will face in the future. It depends on the data to sort out the problems of small firms. If there is no data, there will be no solution. To cope with modern issues, SMEs need a proper data management system to adopt e-commerce and work with it [4].

Small and medium-sized enterprises also use Enterprise Resource Planning (ERP) programs as ICT tools in manufacturing [39]. Technological factors can benefit organizations multiple times. ICT adoption in developing countries is slow, like Bangladesh, where lack of proper telecommunication infrastructure, limited technological literacy, and the inability to merge technological tools into business processes are prominent causes. Also, high costs and limited financial resources for small and medium-sized enterprises cause slower progress of technological tools in the country's businesses [53].

The term "technological factors" denotes three sub-terms: information and communication technology adoption (ICT), which is computerized information; use of the web and e-mail for organizational purposes; and a different type of software used in enterprises [14]. These technologies use desktop and laptop computers, smartphones, handheld devices, the internet, and other business software for data storage and secure networking. Internet Connectivity (IC) supplies quality internet connectivity to their needs, demands, and deliveries. Business data management (BDM) helps organizations and enterprises (SMEs) in the conduction of business processing from various departmental duties that ease employees causing over-burden on work and allow them to deliver timely services [56]. All these processes are carried out over ICT platforms.

This research uses the following definition of SMEs: A micro enterprise is a business with fewer than ten employees. Small enterprises consist of more than ten and less than fifty employees. Medium-sized enterprises consist of more than fifty and less than two hundred employees [40]. SMEs serve ample tasks within supply chains, distributors, and suppliers. Cost reduction is the first benefit of ICT adoption. Previously, the use of paper instead of computers and no proper form of communication or time-taking communication process cost enterprises. In modern times, with the help of ICT, smartphones provide quick and easy communication at any time. Also, the use of computers in firms/organizations has simplified day-to-day tasks, and the processes of financial management, risk management, and business management have become easier and more rapid [36]. If it is considered that there are two types of firms: those using ICT and those not using ICT, then there is no competition between them because, with time, firms will adopt ICT for their benefit. In the case of "competition seeking,' each firm struggles to adopt ICT, but there are several issues in handling the ICT process within organizations.

## Literature review and hypothesis development

### Theoretical background

Small and medium-sized enterprises are a blessing for the growth and development of Bangladesh and its economy [36]. The adoption of e-Commerce in SMEs has changed the perspective of production and innovation in the past few years. Although with progress, some barriers stop the adoption process of e-commerce. These barriers also create hurdles in adopting technological factors, information and communication technologies, and their utilization in firms or enterprises [15]. The barriers include,lack of awareness about the use of information technology, the inadequacy of ICT-capable and literate managers and workers, insufficient financial resources, and the perceived lack of relevance or value-added of ICTs to their business. The adoption of e-commerce depends on research and development activity and productivity improvement [7]. Customers play a vital role in the development of e-commerce. However, technology literacy is still very limited in most developing countries. There is a shortage of skilled workers among SMEs, a key issue in moving forward with using information technology in business [4]. There are also doubts about whether SMEs can take advantage of the benefits of accessing the global market through the Internet, given their limited capabilities in design, distribution, marketing, and post-sale support. While the Internet can be useful in accessing international design expertise, SMEs are not confident that they can command a premium on the prices of their goods unless they offer product innovations [65].

Through the emergence of technology, many businesses today can be wider and more expansive in the business environment by adopting e-Commerce. A more expansive and extensive form of technological advancement through Information and Communication Technology adoption (ICT) helps many Small and Medium Sized Enterprise (SMEs) to conduct businesses through the integration of different forms of communication lines that will connect the business to customers at their fingertips and will be exposed to business information regarding products and services [3, 10, 14]. An array of studies has been conducted on the topics of e-commerce adoption and ICT adoption. Many of these studies focused on developed countries, while fewer focused on developing countries [18, 24, 30]. Studies carried out in developing countries focused on the downstream side of SMEs rather than the upstream side, which makes the previous literature unidirectional concerning ICT adoption in SMEs. There is a need to develop factors that impact e-commerce adoption in small firms and understand the role of information and communication technology as per modern standards. However, business performance is important and needs to utilize these factors to benefit the organization and firms [10]. Major problem faced by SMEs in the adoption of e-commerce is the lack of information technology [42]. In developing countries, ICT adoption does not provide enough benefit to SMEs, which is the main reason to keep ICT out of focus [33].

One important aspect of e-Commerce is the technological concept of providing an organization with ease of doing business, which is especially helpful in many small and medium-sized enterprises (SMEs) across the globe because of its various advantages for effective and efficient business outcomes. Several factors in e-commerce adoption don't support the adoption of e-commerce in SMEs [7]. These factors include supportive issues in the form of lack of support, lack of security measures, business partner readiness, and lack of information technology resources. These are the major factors in the adoption of B2C e-commerce. Other factors Zaied [68] listed are the lack of proper knowledge and usage ability of the ICT resources, no maintenance of the ICT equipment, improper communication infrastructure, financial issues, and legal infrastructure. Although SMEs play an important role in developing the country's economy and growth. In some cases, the country's economic growth depends on small and medium-sized firms, which help create jobs and eradicate poverty. There, there is always a positive impact in terms of the economic growth of the country with the development of SMEs [13, 67].

### ICT adoption by SMEs

Research conducted by [31] to examine the role of ICT in business development concluded that ICT's role in business growth is positive and productive. ICT has played a major role in the development of organizations, especially by facilitating communication, through which firms or organizations transfer valuable data in a second [48]. Also, ICT has advanced the process of information gathering with a single click. In times of need, ICT helps organizations manage their production on time. The enterprises that use information technology are more productive than others. The growth is double, and they grow faster. From the profit point of view, ICT-adopted firms/organizations always earn a better output [32]. Pandemic shifted the entire focus of businesses in 'business survival. In pandemic, consumer's attention was shifted to compatibility, and the perceived complexity remained high in e-commerce adoption [23]. Compared with other organizations, ICT-adopted firms encourage other firms to adopt the same mechanism. Small enterprises don't use ICT in their business. The reasons are the lack of proper information, small output, and considering ICT as a complex and insecure tool. Another reason is the lack of skills in SMEs to use ICT [41]. Based on the literature review, the hypothesis of the research is,

H1. ICT adoption has positive impact on SMEs e-commerce adoption.

#### Internet connectivity (IC)

The internet connection is the first demand for small and medium-sized enterprises to adopt e-commerce. The internet not only connects SMEs on equal grounds but also connects the firms with international markets. Internet-based information and communication technologies provide more value to businesses through quick interaction, access to data, and new modes of formulating a business. According to [22, 31, 47], ICT has improved the communication between SMEs and competition among firms. ICT is information oriented, enabling the firm to access information directly from the stakeholder. ICT is communication-oriented, which enables SMEs to connect with stakeholders in multiple ways. Also, the ICT provides workflow orientation for automated processing. Balocco et al. [8] argue that proper ICT usage not only lowers the costs of communication but also eradicates the barriers to communication, allowing for a rapid increase in the exchange of information from one stakeholder to another. Information flow is the demand of today's small and medium firms, and internet connectivity fulfills the demand. Businesses more attached to ICT are more progressive and have better results than those without internet access. Internet connectivity has benefited organizations and firms at the root level and eradicated the traditional means of communication [21]. Taking orders, keeping a record of product delivery, and providing feedback mechanisms are all done through internet connectivity, which eases the link between different branches of SMEs. Based on the literature review, the hypothesis of the research is,

H2. Internet Connectivity has positive impact on SMEs e-commerce adoption.

#### Business data management (BDM)

Nowadays, data for small and medium-sized enterprises are considered the biggest asset rather than products. Products are not unique and have background data, which is the main asset. Enterprises are exploring unique ways to cultivate and store the data for longer periods. In today's world, data has become bigger, and big data management in business is an issue. SME participants go through different stages to mark their venture for business data management [64]. In the first place, they seek a potential business partner. The business partner provides the firm with their basic demands through the supply chain process, in which the suppliers of the products etc. [63, 70]. After completion of the first task, a mutual agreement is needed for all the partners to meet their demands. In this phase, the price of products, the time of product delivery, and the cost of product delivery are negotiated. The second phase is about the practical agreement of the first phase. In all these phases, business data management is needed.

There are ample phases, including the research and R&D phases [61]. Multinational firms use big data, but SMEs have also progressed to big data management. SMEs acquire ample data on products, planning, and tools for improving business functionality. According to [25], big data achievement is not an issue for SMEs,rather, the issue is managing it. SMEs utilize their power through partnership programs, logistics, and customer-based demands. There are many challenges for SMEs, and big data provides a valuable solution. The key is successful data management. Data management has challenges for SMEs regarding security issues, handling the different types of data simultaneously, extracting value from the data, data analysis, and data storing [49]. Based on the literature review, the hypothesis of the research is,

H3. Business data management has positive impact on SMEs e-commerce adoption.

### E-Commerce adoption factors

The e-commerce adoption in this paper has three factors, (1) Strategic innovation, (2) Research and development activity, and (3) Productivity growth.

#### Strategic innovation (SI)

In previous studies, three stands have been discussed relating to the relationship between productivity growth and strategic innovation for SMEs. The first stand has been discussed by [20] in the *capital stock model*. This model highlights the investment in research and development activity through strategic innovation. Other studies also focused on strategic innovation in terms of productivity growth. [27] highlighted the use of "knowledge capital" as the main source of strategic innovation. According to Smolny [58], strategic innovation improves productivity by reducing production costs and capital in the third model. Another model of active learning introduced in the studies [59] points out the successful contribution of strategic innovation to the firm's productivity. Studies from the USA highlight the strategic innovation process as an essential element in the growth of firm innovation [37]. The R&D researchers conducted these studies on practical grounds to inspect the SME' single product venture. Companies have been observed to go for a single famous product to secure their best place in the market and highlight the available competition with other products on the ground [5, 12]. Based on the literature review, the hypothesis of the research is;

H4. Strategic innovation has positive impact on SMEs e-commerce adoption.

#### Research and development activity (RDA)

It has been observed in previous studies that small and medium-sized firms only limit themselves to informal research and development activity, which is the main reason for their downward bias, which further leads to their limited standard of innovation [55]. Research and development activities must be permanent for SMEs. Still, in the case of small and medium-sized firms, non-permanent research and development are observed with the attachment of different departments [2]. Innovation activities grow in SMEs where limited interaction with the government is involved, leading to higher responsiveness. Research and development activity is the keys to creating change in the technological process [46]. In challenging times, firms try to maintain their capacity by maintaining reliability for continuous growth. In that case, the business improvement along with the manufacturing process is the key to ensuring the firm's sustainable growth. According to [45], managers in small and medium firms have lesser skills to lead the company strategically. SMEs serve the local niche with limited skills to produce a product that benefits the limited community. Resource scarcity is another barrier to SMEs' limited innovation with less organizational structure, a lack of expert employees, skillful workers, and proper knowledge about product creativity and innovation [16]. Developing new market-competitive products comes through SMEs' research and development activity [57]. The competition of SMEs is through their new-to-market products. In this regard, research and development are the keys to creating a product design and ensuring the product's reliability and demand in the market [28]. In most cases, R&D activity is helpful in the greater growth of the industry. However, small firms invest more in R&D at the local level with limited resources. Based on the literature review, the hypothesis of the research is,

H5. Research and Development has positive impact on SMEs e-commerce adoption.

#### Productivity growth (PROD)

The effect of e-commerce adoption on the supply chain and demand is obvious in SMEs. It has a profound impact on consumer behavior relating to economic development. On the demand side, it increases productivity growth and influences the production treatment on the supply level. For most factors, there is a well-established relationship between e-commerce and productivity growth [6]. In some previous studies, the relationship between economic growth and productivity growth has been observed regarding ICT adoption. Many studies show a positive relationship between productivity growth and ICT adoption at different levels. It has been observed that ICT can increase the level of productivity growth for SMEs. According to [69], e-commerce adoption has led firms to sustain better economic growth and the development of small firms. However, productivity growth results from different factors that coordinate to obtain sustainable growth in the firm [1, 26]. In low-development countries, e-commerce has always been a strong force behind the progressive economy. Based on the literature review, the hypothesis of the research is,

H6. Productivity Growth has positive impact on SMEs e-commerce adoption.

### Conceptual framework

The technology facilitating the communication process in the industry is called Information and Communication Technology (ICT) [41]. In today's firms, the role of ICT is to facilitate small and medium-sized enterprises with computer networks and telecommunications, which further facilitate broadband technology for the industries. According to the World Bank, ICT helps manage the hardware, software, and networks to collect and store the data for transmission and processing purposes [32]. It also helps the industry to provide voice, text, and images. The terms "information technology" and " information and communication technology" have the same purpose. The literature review has drawn the research framework, highlighting the utilization of technological factors by SMEs in Bangladesh and its impact on e-Commerce adoption. Technological factors include (1) information and communication technology adoption (ICT), which further includes (2) internet connectivity, and (3) business data management [17]. Other factors relating to e-commerce adoption are (1) strategic innovation, (2) research and development activity, and (3) productivity growth. ICT adoption is not limited to a few factors. It includes access to the internet, computers, printers, scanners, smartphones, and other devices to fulfill the SMEs' basic needs. The benefit of ICT adoption is significant. However, there are several barriers to implementing ICT adoption in small and medium-sized enterprises [4]. The use of ICT and its impact on e-Commerce adoption in SMEs has been observed in previous studies. In Bangladesh, the lack of proper infrastructure and internet connectivity is an issue for SMEs to grow rapidly, negatively impacting e-commerce adoption [1]. This research explores the factors behind SMEs' e-commerce adoption in Bangladesh. The factors have been drawn through a literature review of the relevant studies. There are unique factors that the previous research hasn't addressed. Also, the previous research focused more on developing e-commerce adoption in developed countries. Studies available on the adoption of SMEs e-commerce in developing countries are conducted upstream. This study explores the downstream issues relating to e-commerce adoption in Bangladesh by small and medium-sized firms. This research includes the following factors: Technological factors such as information and communication technology adoption (ICT), internet connectivity (IC), and business data management (BDM) are included. The e-commerce adoption factors are strategic innovation (SI), research and development activity (RDA), and productivity growth (PG).

## Methodology and sample size

Research strategy is the most basic and common approach in management and business research [52]. This pattern of research aims to collect data from samples. Data is used through descriptive statistics for research purposes based on deductive logic [52]. This research collected data from SMEs in Bangladesh with self-administered questionnaires. It is a 5-point Likert scale questionnaire based on six technological factors and e-commerce adoption. Other empirical previous studies have used the same mechanism to identify key constructs of research variables. The questionnaire deals with the demographic profile of the respondents, which includes the respondents' age, Gender of the respondents, their position in small and medium firms, and how long the SMEs have been working. The technological factors include ICT adoption, Internet Connectivity, and Business Data Management to explore their impact on SMEs' ability to adopt ICT. The e-commerce adoption factors are,Strategic innovation, research and development activity, and Productivity growth.

This study uses a method to enlist SMEs with fewer than 100 employees. In Bangladesh, there is no official data available over the internet relating to small and medium-sized firms. The random selection method has been advised to collect data from SMEs aged not less than five years. The data collected for this research is through the interested parties of small and medium sized enterprises in Bangladesh. Only those firms were selected for data collection having completion information, record available to the researcher. Complete information of relevant small and medium sized enterprises includes the company name, address, e-mail address, industry type, and contact number. The data of the research is collected through a questionnaire. The questionnaire of the research was prepared by the researcher, based on the literature review of the relevant previous studies. After identifying and cleaning the data, 115 small and medium-sized firms were selected to collect data with 350 respondents.

**Fig. 1:**
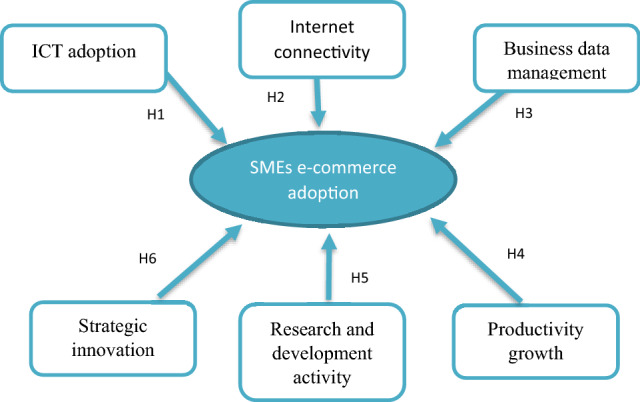
Research framework, technological factors, SMEs and e-commerce factors

In the data analysis procedure, the descriptive statistics of the research variables is given with their Mean and Standard deviation. The KMO and Bartlett's test is applied to examine the reliability and validity of the construct. Factor analysis process has been completed to examine the total variance explained. The co-relation and regression process is performed for hypothesis investigation (Fig. 1).

## Data analysis

The demographic profile of the respondents is based on the age of the respondents, the position of employees in small and medium sized enterprises in Bangladesh, and How long the business of SMEs is running (Table 1).

**Table 1:** Demographic profile

		% of total
Gender	Male	78.3
	Female	21.7
Age	18–24 years	1.1
	19–27 years	50.0
	28–34 years	34.3
	Over 35 years	14.6
Position in SME	Owner	12.6
	Manger	10.9
	Both	9.4
How long has the business been running for?	Less than 5 Years	15.1
	6–10 years	9.7
	11–25 years	5.7
	Over 25 years	2.3

The demographic profile of the respondents shows that 78% of the employees are male, and 21% of employees are female in SMEs. 50% of the respondents have an age range of 19–27 years, 34% of their age range is 28–34 years, and the least number of respondents have an age below 24 years. Regarding the position of employees in SMEs, 12% were owners of SMEs, 10% were managers, and 9% were both managers and owners. The business has been running for less than five years and has a ratio of 15%, while 9% of the business is over 6 years of age and below 10 years, while over 25 years of business has a ratio of 2.3%.

### Descriptive statistics of the variables

This section descriptive statistics of the results shows the Mean and Standard deviation (Tables 2, 3).

**Table 2:** Descriptive statistics

	Mean	N	SD
SMEs e-commerce adoption	4.64	350	.481
ICT adoption	4.6114	350	.55410
Internet connectivity	4.6743	350	.51026
Business data management	4.6457	350	.49660
Research and development activity	4.0071	350	.70045
Strategic innovation	4.3300	350	.52484
Productivity growth	4.1586	350	.49662

**Table 3:** KMO and Bartlett's test

Kaiser–Meyer–Olkin measure of sampling adequacy		.753
Bartlett's Test of Sphericity	Approx. Chi-Square	3097.389
	df	190
	Sig	.000

Kaiser Meyer's measurement adequacy is 0.753, which is greater than 0.6 to assure us that we can start with an exploratory factor analysis process. Also, Bartlett's test of sphericity is significant (Table 4).

**Table 4:** Total variance explained

Component	Initial Eigenvalues			Extraction sums of squared loadings			Rotation SS Loadings^a^
	Total	Variance %	Cumulative %	Total	Variance %	Cumulative %	Total
1	4.892	24.458	24.458	4.892	24.458	24.458	4.135
2	2.799	13.993	38.451	2.799	13.993	38.451	2.349
3	1.671	8.357	46.808	1.671	8.357	46.808	2.026
4	1.498	7.489	54.296	1.498	7.489	54.296	2.363
5	1.379	6.894	61.191	1.379	6.894	61.191	2.502
6	1.232	6.159	67.350	1.232	6.159	67.350	2.592

Extraction Method: Principal Component Analysis. a. When components are correlated, sums of squared loadings cannot be added to obtain a total variance

The initial Eigenvalues table shows that we have seven components having eigenvalues above than 1. It further means that in our study, we have 6 factors.

Component Matrix in the Table 5 is the rotation-based matrix of factor analysis. The extraction method used in the factor analysis is ‘Principal component analysis’, which extracted six components.

**Table 5:** Component Matrix^a^

	Component					
	1	2	3	4	5	6
Internet connectivity	.845					
ICT adoption		.805				
Research and development activity			.729			
Productivity growth				.710		
Strategic innovation					.536	
Business data management						.480

Extraction Method: Principal Component Analysis

a. 6 components extracted

A Pearson correlation was conducted to determine the relationship between variables of e-commerce adoption and technological factors. The study results show a significant relationship with SME’s e-commerce adoption. In the case of strategic innovation, the relationship is non-significant (r = 0.336, *p* < 0.05). In contrast, the ICT adoption (r = 0.409), Internet connectivity (r = 0.327), Business data management (r = 0.256), research and development activity (r = 505), and productivity growth (r = 0.552). The results show that productivity growth (PG) and research and development activity (RD) strongly relate to SMEs e-commerce adoption (Tables 6, 7).

**Table 6:** Co-relation of the research variables

Correlations			
	Pearson Correlation	Mean	p
ICT adoption	.409	4.6114	0.000
Internet Connectivity	.327	4.6743	0.000
Business Data Management	.256	4.6457	0.000
Research and Development activity	.505	4.0071	0.000
Strategic innovation	.336	4.3300	.369
Productivity Growth	.552	4.1586	0.000

**Table 7:** Result of the regression

Coefficients^a^				
	B	Std. Error	t	Sig
(Constant)	.939	.240	3.904	.000
ICT adoption	.184	.045	2.880	.031
Internet Connectivity	−.016	.047	−.336	.737
Business Data Management	.004	.042	.105	.916
Research and Development activity	.253	.028	9.091	.000
Strategic innovation	.200	.039	5.165	.000
Productivity Growth	.358	.043	8.308	.000

^a^Dependent Variable: e-Com adoption by SMEs

R = 0.706 R^2^ = .499 F = 56.9 Sig. = .000

A regression analysis was conducted to explore the impact of technological and e-commerce factors on SMEs e-commerce adoption. The results of the regression are given in the table. The value of R shows a simple correlation among variables. In this case, the R-value (R = 0.706) indicates a higher correlation level among variables. The R square value shows how much of the total variance is explained by the dependent variable. In this case, the value of (R^2^ = 0.499) indicates that 49% of the variance is explained by SMEs e-commerce adoption. In the case of internet connectivity (IC), the value of β is negative (β = −0.016, p > 0.05), which shows a non-significant negative relationship between SMEs e-commerce adoption and internet connectivity. Business data management has a non-significant impact on SMEs e-commerce adoption (β = 0.004, *p* < 0.005).

Table 8 addresses the hypothesis statements and their results in the status sections. This research is based on six hypotheses, of which four hypotheses; (H1) ICT adoption, (H4) productivity growth, (H5) Research and development activity, and (H6) strategic innovation, has been accepted by the results. The rejected hypotheses of the study are; (H2) internet connectivity and (H3) business data management.

**Table 8:** Hypothesis results

	Statement	Status
H1	ICT adoption has positive impact on SMEs e-commerce adoption	Accepted
H2	Internet Connectivity has positive impact on SMEs e-commerce adoption	Rejected
H3	Business data management has positive impact on SMEs e-commerce adoption	Rejected
H4	Productivity Growth has positive impact on SMEs e-commerce adoption	Accepted
H5	Research and development activity has positive impact on SMEs e-commerce adoption	Accepted
H6	Strategic innovation has positive impact on SMEs e-commerce adoption	Accepted

## Discussion

This study evaluated the "utilization of technological factors to promote the e-commerce adoption in SMEs". The study results show that productivity growth, research and development activity strongly effect the e-commerce adoption in small and medium enterprises in Bangladesh. To examine, either the effect is positive or negative, regression analysis report shows; a negative relationship between internet connectivity and SMEs e-commerce adoption. Internet connectivity is the backbone to any e-commerce enterprise all over the globe. The negative effect shows that; if there is no internet connectivity, there will be no e-commerce business. According to Taneja [62] the rising demand of e-commerce business has increased the demand of power, electricity, transportation, computer hardware, software and continuous internet connectivity. This study proves the strong effect of internet connectivity with e-commerce adoption. A similar case is observed by Mohdhar and Shaalan [38] the future internet connectivity to 5G will revolutionize the e-commerce industry with meta universe conception of online selling and buying of products, in which the consumer can avail the opportunity to feel the product over the meta platforms. This initiative has already been in progress through Facebook platform. The internet connectivity is the key to the e-commerce operations of the firms. In Bangladesh, the online internet was legalized in 1996. Information services Network (ISN) provided internet in Bangladesh. Later the internet service providers (ISP) increased to Grameen Cybernet and non-governmental organizations working with NGOs. The recent developments of G7 summit on the information technology adds that increasing information technology will integrate the developing countries to the global economy, that will enable these countries to develop their own infrastructure. However, Bangladesh is not internet barrier free country. Poor internet connectivity is common phenomenon in Bangladesh. Environmental issues, bad weather has negative impact on the internet connectivity in Bangladesh. Slow internet has always been an issue to be resolved in Bangladesh for small and medium sized enterprises.

Every small and medium-sized firm needs data. Data is the key to the progressive future of the industry. If the SME has limited access to data, or there is no access to data, it has no competition with other firms globally. Data resolves conflicts between firms. Also, it helps generate new ideas [50]. In cases where the data is the key, there is a need to store the data. Data is becoming bigger and bigger day by day [43]. The data is delivered to the products available on the website of the small and medium-sized firms. It also contains data relating to the demographics of the consumers. Their details of shopping, their experience, and the customers' credit details are the data. Also, the data of the past is equally important for SMEs. Big data storage also creates an issue for SMEs.

There is insignificant relationship between Business data management and SMEs e-commerce adoption shows the concern relating to data management in business for small and medium firms in Bangladesh. However, this conflict can be resolved by increasing safe internet connectivity and proper data storage functionality [9]. Also, data analysts are needed to interpret the data, which takes time and effort. There is no easy way for SMEs to get value out of data. Research and development have a weak relationship with SMEs e-commerce adoption, which shows a lack of research and development activity in SMEs. In small firms, where data is limited and the scope is also limited, there is a dire need for R&D [29]. A study conducted by Yan et al. [66] shows the acquisition technology of transactional information has strong impact on the accurate collection of data for small and medium sized enterprises working over e-commerce platforms.

In the research and development sector the role of small and medium sized enterprises is revolutionizing and truing it into big enterprises. SMEs struggle more in research and development than big firms because the struggling SMEs in developing countries continuously explore new factors to emerge as vibrant products in the market. They are using tools available on board. These tools and ideas need proper functionality in research and development. However, the lack of knowledge, lack of information, and lack of expertise is the barrier to research and development. Data is needed to practice the proper research in SMEs. A study conducted by Sumahir et al. [60] shows the impact of e-commerce adoption on research and development in Hungary, Poland and Slovenia. The results of the study shows that e-commerce adoption without adopting the R&D cannot lead the future industries. Also, the R&D sector empower the e-commerce and revolutionize it with new sources of information.

Strategic innovation and productivity growth have a significant relationship with each other. Also, the strategic innovation positively effects the SMEs e-commerce adoption. The similar results have been drawn by Alraja et al., [3] and Muangmee et al., [40] that strategic innovation introduces new ideas to empower the e-commerce industry. The results of the study show, productivity growth also has a significant relationship with product innovation. The similar results have been drawn by in a previous research Ale Ebrahim et al., [2] and Smolny, [59]. Productivity growth is similar to the growth factor in SMEs. Still, it has a different view when classified as the overall growth of SMEs by contributing positive value to the country's economy. The positive contribution is considered valid when small and medium-sized firms explore their concerns less negatively and more positively. In some cases, there is no need to notice the impact of the government or environment on the SMEs growth factor. The government continuously changes with positive or negative changes in SMEs policy-making [6].

## Conclusion

The adoption of information and communication technology (ICT) in Bangladesh is very interesting for small and medium-sized enterprises. The COVID-19 pandemic was a giant hit for the world, in which developing countries faced severe lockdown and seizure of businesses and markets for months. In the meantime, consumers and producers were eager to adopt ICT to run their businesses, and customers willingly sought ways to buy products online. This transformation has enlarged the e-commerce businesses. Small and medium firms adopted ICT, significantly impacting e-commerce adoption in Bangladesh. Hypothesis H1 addresses the same case of ICT adoption positively impacting SME's e-commerce adoption. To adopt e-commerce, firms need internet connectivity, which positively impacts SME's e-commerce adoption in Bangladesh as per Hypothesis H2. This hypothesis was rejected on certain grounds, which doesn't mean that internet connectivity has a negative impact on e-commerce adoption, but internet availability is limited for SMEs in Bangladesh. Small and medium-sized firms have less access to speedy internet services. Also, the internet service providers struggle to expand their coverage to every area of Bangladesh. As is the case with business data management, which has a pivotal role in adopting e-commerce for SMEs. Data management is a job of expertise; putting value out of data is a skill. SMEs in Bangladesh lack expertise in data management. However, data provides the solution to every problem of SMEs. More data, more chances to solve issues. Hypothesis H3 addresses whether business data management positively impacts e-commerce adoption, which is rejected in the research findings. Productivity growth is the increment in the output of the production of SMEs. Productivity growth in SMEs is linked to the total output. In this research, hypothesis H4, Productivity Growth, has a positive impact on SMEs e-commerce adoption, which has a significant positive impact on SME's e-commerce adoption.

Small and medium-sized firms have adopted a normal level of productivity growth in Bangladesh. Research and development (hypothesis H5) have a strong positive impact on SME's e-commerce adoption. The same is the case with hypothesis H6. Strategic innovation positively impacts SME's e-commerce adoption. Strategic innovation, research & development are the backbones of any SMEs in developing countries. R&D has equal value for every firm, whether small or multinational. It has been observed that small industries struggle more in R&D, which is equally appreciated in multinational firms. However, the scope of strategic innovation in SMEs is limited according to the demand of the SMEs. Small firms work locally and have a limited scale in strategic innovation. On the other hand, multinational firms have larger-scale strategic innovations. The study results are equally related to the literature review and, in comparison, to the hypothesis drawn from the review of the relevant literature.

These findings of the study are helpful for the local government policy makers in Bangladesh in terms of e-commerce promotion. This finding is provided insight for the central government to enhance the e-commerce setup in Bangladesh by facilitating the SMEs with updated information and knowledge on Information technology, IT skills so that micro level enterprises can empower themselves with proper usage of social media strategies. This finding contains the information relating to data management, and it helps the government sector as well as private sector to enhance the access to data.

This study contributes the evidence of research and development activity growth in the industry. Also, it links the productivity growth and internet availability to the SMEs in every region of Bangladesh. This finding is useful as reference for the academic and researchers on the same model.

## References

[1] Akhter A, Karim MM, Jannat S, Islam KA (2022). Determining factors of intention to adopt internet banking services: A study on commercial bank users in Bangladesh. Banks and Bank Systems.

[2] Ale Ebrahim N, Ahmed S, Taha Z (2010). SMEs; Virtual research and development (R&D) teams and new product development: A literature review. International Journal of the Physical Sciences.

[3] Alraja MN, Imran R, Khashab BM, Shah M (2022). Technological innovation, sustainable green practices and SMEs sustainable performance in times of crisis (COVID-19 pandemic). Information Systems Frontiers.

[4] Amornkitvikai, Y., Tham, S. Y., & Tangpoolcharoen, J. (2021). Barriers and factors affecting E-commerce utilization of Thai small and medium-sized enterprises in food and beverage and retail services. *Global Business Review,* 09721509211036294.

[5] Andersen TCK, Aagaard A, Magnusson M (2022). Exploring business model innovation in SMEs in a digital context: Organizing search behaviours, experimentation and decision-making. Creativity and Innovation Management.

[6] Anvari RD, Norouzi D (2016). The impact of e-commerce and R&D on economic development in some selected countries. Procedia-Social and Behavioral Sciences.

[7] Ariansyah K, Sirait ERE, Nugroho BA, Suryanegara M (2021). Drivers of and barriers to e-commerce adoption in Indonesia: Individuals’ perspectives and the implications. Telecommunications Policy.

[8] Balocco, R., Mogre, R., & Toletti, G. (2009). Mobile internet and SMEs: A focus on the adoption. *Industrial Management & Data Systems*.

[9] Bappy, A. (2018). *E-commerce business opportunities and challenges in Bangladesh*.

[10] Billal HM, Shin HK, Sim WJ (2019). Critical success factors (CSF) on e-commerce adoption in Bangladesh SMEs. Management Review: An International Journal.

[11] Bosnjak M, Ajzen I, Schmidt P (2020). The theory of planned behavior: Selected recent advances and applications. Europe's Journal of Psychology.

[12] Carrasco-Carvajal O, Castillo-Vergara M, García-Pérez-de-Lema D (2022). Measuring open innovation in SMEs: an overview of current research. Review of Managerial Science.

[13] Chua XJ, Kasim N, Zainal R, Musa SMS (2022). ICT implementation for improving communication among SME contractors in construction industry. Research in Management of Technology and Business.

[14] Chung CJ, Nam Y, Stefanone MA (2012). Exploring online news credibility: The relative influence of traditional and technological factors. Journal of computer-mediated communication.

[15] Costa J, Castro R (2021). SMEs must go online—E-commerce as an escape hatch for resilience and survivability. Journal of Theoretical and Applied Electronic Commerce Research.

[16] Elhusseiny HM, Crispim J (2022). SMEs, Barriers and Opportunities on adopting Industry 4.0: A Review. Procedia Computer Science.

[17] Erumban AA, De Jong SB (2006). Cross-country differences in ICT adoption: A consequence of Culture?. Journal of world business.

[18] Fatoki O, Asah F (2011). The impact of firm and entrepreneurial characteristics on access to debt finance by SMEs in King Williams' town, South Africa. International Journal of Business and management.

[19] Ghobakhloo, M., Arias‐Aranda, D., & Benitez‐Amado, J. (2011). Adoption of e‐commerce applications in SMEs. *Industrial Management & Data Systems*.

[20] Griliches Z (1979). Issues in assessing the contribution of research and development to productivity growth. The Bell Journal of Economics.

[21] Guerriero M (2015). The impact of Internet connectivity on economic development in Sub-Saharan Africa. EPS Peaks.

[22] Harrigan P, Schroeder A, Qureshi I, Fang Y, Ibbotson P, Ramsey E, Meister D (2010). Internet technologies, ECRM capabilities, and performance benefits for SMEs: An exploratory study. International journal of electronic commerce.

[23] Hossain MB, Wicaksono T, Nor KM, Dunay A, Illes CB (2022). E-commerce adoption of small and medium-sized enterprises during COVID-19 pandemic: Evidence from South Asian Countries. The Journal of Asian Finance, Economics and Business.

[24] Hu Q, Mason R, Williams SJ, Found P (2015). Lean implementation within SMEs: A literature review. Journal of Manufacturing Technology Management.

[25] Iqbal, M., Kazmi, S. H. A., Manzoor, A., Soomrani, A. R., Butt, S. H., & Shaikh, K. A. (2018). A study of big data for business growth in SMEs: Opportunities & challenges. *Paper presented at the 2018 International conference on computing, mathematics and engineering technologies (iCoMET)*.

[26] Islam A, Wahab S, Latiff A (2022). Annexing a smart sustainable business growth model for small and medium enterprises (SMEs). World Journal of Entrepreneurship, Management and Sustainable Development.

[27] Klette J, Førre SE (1998). Innovation and job creation in a smallopen economy-evidence from norwegian manufacturing plants 1982–92. Economics of Innovation and New Technology.

[28] Kock H, Ellström PE (2011). Formal and integrated strategies for competence development in SMEs. Journal of European Industrial Training.

[29] Kumar M, Ayedee D (2021). Technology adoption: A solution for SMEs to overcome problems during COVID-19. Forthcoming, Academy of Marketing Studies Journal.

[30] Kurnia S, Choudrie J, Mahbubur RM, Alzougool B (2015). E-commerce technology adoption: A Malaysian grocery SME retail sector study. Journal of Business Research.

[31] Kyakulumbye S, Pather S (2022). Understanding ICT adoption amongst SMEs in Uganda: Towards a participatory design model to enhance technology diffusion. African Journal of Science, Technology, Innovation and Development.

[32] Kyobe M (2011). Investigating the key factors influencing ICT adoption in South Africa. Journal of Systems and Information Technology.

[33] MacGregor RC, Kartiwi M (2010). Perception of barriers to e-commerce adoption in SMEs in a developed and developing country: A comparison between Australia and Indonesia. Journal of Electronic Commerce in Organizations (JECO).

[34] Marangunić N, Granić A (2015). Technology acceptance model: A literature review from 1986 to 2013. Universal Access in the Information Society.

[35] Davis, F.D. (1986). A technology acceptance model for empirically testing new end-user information systems: theory and results. Doctoral dissertation. MIT Sloan School of Management, Cambridge, MA.

[36] Mendy J (2021). Performance management problem of four small and medium-sized enterprises (SMEs): Towards a performance resolution. Journal of Small Business and Enterprise Development.

[37] Miles MP, Covin JG (2002). Exploring the practice of corporate venturing: Some common forms and their organizational implications. Entrepreneurship theory and practice.

[38] Mohdhar, A., & Shaalan, K. (2021). The future of e-commerce systems: 2030 and beyond. In *Recent Advances in Technology Acceptance Models and Theories* (pp. 311–330). Springer.

[39] Moon, Y. (2007). *Enterprise Resource Planning (ERP): A review of the literature*.

[40] Muangmee C, Dacko-Pikiewicz Z, Meekaewkunchorn N, Kassakorn N, Khalid B (2021). Green entrepreneurial orientation and green innovation in small and medium-sized enterprises (SMEs). Social Sciences.

[41] Mushtaq R, Gull AA, Usman M (2022). ICT adoption, innovation, and SMEs’ access to finance. Telecommunications Policy.

[42] Narantsetseg C, Dunay A, Nyamdulam T (2021). The Role Of Information Technology For Small Medium Enterprises: Focusing On Vehicle Companies In Mongolia. International Journal of Social Science and Humanities Research-MIYR.

[43] Nusrat, M., & Sultana, N. (2019). Soft skills for sustainable employment of business graduates of Bangladesh. *Higher Education, Skills and Work-Based Learning*.

[44] Orr, G. (2003). Diffusion of innovations, by Everett Rogers (1995). Retrieved January, 21, 2005.

[45] Ortega-Argilés R, Vivarelli M, Voigt P (2009). R&D in SMEs: A paradox?. Small Business Economics.

[46] Pandya, V. M. (2012). Comparative analysis of development of SMEs in developed and developing countries. *Paper presented at the The 2012 International Conference on Business and Management*.

[47] Park S (2022). Success Stories of ICT Adoption by Nigerian SMES During the Covid-19 Pandemic. International Journal of Business and Management Sciences.

[48] Pellegrina LD, Frazzoni S, Rotondi Z, Vezzulli A (2017). Does ICT adoption improve access to credit for small enterprises?. Small Business Economics.

[49] Quix C, Schoop M, Jeusfeld M (2002). Business data management for business-to-business electronic commerce. ACM SIGMOD Record.

[50] Rattanawiboonsom V, Ali MM (2016). Factors affecting entrepreneurial management in Bangladesh: an empirical analysis. Problems and Perspectives in Management.

[51] Roy S (2019). Industrial Policy 2016 of Bangladesh: An Assessment from the Green Perspective. Bangladesh Journal of Public Administration (BJPA)..

[52] Saunders, M., Lewis, P., & Thornhill, A. (2007). *Research methods. Business Students* (4th edn). Pearson Education Limited.

[53] Shiels H, McIvor R, O'Reilly D (2003). Understanding the implications of ICT adoption: Insights from SMEs. Logistics information management.

[54] Silva, P. (2015). Davis' technology acceptance model (TAM) (1989). *Information seeking behavior and technology adoption: Theories and trends* (pp. 205–219).

[55] Singh, R. K., Garg, S. K., & Deshmukh, S. (2008). Strategy development by SMEs for competitiveness: A review. *Benchmarking: An International Journal*.

[56] Singh, S., & Singh, J. (2022). *A survey on master data management techniques for business perspective cyber intelligence and information retrieval* (pp. 609–617). Springer.

[57] Smith H, Discetti R, Bellucci M, Acuti D (2022). SMEs engagement with the Sustainable Development Goals: A power perspective. Journal of Business Research.

[58] Smolny W (1998). Innovations, prices and employment: A theoretical model and an empirical application for West German manufacturing firms. The Journal of Industrial Economics.

[59] Smolny W (2000). Sources of productivity growth: An empirical analysis with German sectoral data. Applied Economics.

[60] Sumahir GN, Wahyudi H, Nirmala T (2022). The Effect of Research and Development (R&D) Investment, E-Commerce Company Employee, and E-Commerce Transaction Volume on Economic Growth in Indonesia 2010Q1–2020Q4. Peradaban Journal of Economic and Business.

[61] Sun, Y., Shi, Y., & Zhang, Z. (2019). *Finance big data: Management, analysis, and applications* (vol. 23, pp. 9–11): Taylor & Francis.

[62] Taneja, B. (2021). The digital edge for M-commerce to replace E-commerce. In *Emerging Challenges, Solutions, and Best Practices for Digital Enterprise Transformation* (pp. 299–318). IGI Global.

[63] Thuraisingham, B. (2000). *Web data management and electronic commerce*. CRC Press.

[64] Willetts, M., Atkins, A., & Stanier, C. (2022). *Quantitative study on barriers of adopting big data analytics for UK and Eire SMEs data management, analytics and innovation* (pp. 349–373): Springer.

[65] Yadav H, Soni U, Gupta S, Kumar G (2022). Evaluation of barriers in the adoption of E-commerce technology in SMEs: A fuzzy DEMATEL approach. Journal of Electronic Commerce in Organizations (JECO).

[66] Yan, B., Wu, C., Yu, R., Yu, B., Shi, N., Zhou, X., & Yu, Y. (2021). *Big data-based E-commerce transaction information collection method. Complexity, 2021*.

[67] Yao, C., Peng, X., Kurnia, S., & Rahim, M. M. (2022). *Understanding Factors Affecting the Adoption of ICT-Enabled Sustainable Supply Chain Management Practices*. Paper presented at the HICSS.

[68] Zaied ANH (2012). Barriers to e-commerce adoption in Egyptian SMEs. International Journal of Information Engineering and Electronic Business.

[69] Zatonatska, T., & Novosolova, V. (2017). Modeling of impact of e–commerce on economic development. *Фiнaнcoвo-кpeдитнa дiяльнicть: пpoблeми тeopiї тa пpaктики(1)*, 265–273.

[70] Zide O, Jokonya O (2022). Factors affecting the adoption of Data Management as a Service (DMaaS) in Small and Medium Enterprises (SMEs). Procedia Computer Science.

